# Rhythmic Temporal Expectation Boosts Neural Activity by Increasing Neural Gain

**DOI:** 10.1523/JNEUROSCI.0925-19.2019

**Published:** 2019-12-04

**Authors:** Ryszard Auksztulewicz, Nicholas E. Myers, Jan W. Schnupp, Anna C. Nobre

**Affiliations:** ^1^Department of Biomedical Sciences, City University of Hong Kong, Hong Kong Special Administrative Region of the People's Republic of China,; ^2^Max Planck Institute for Empirical Aesthetics, 60322 Frankfurt am Main, Germany,; ^3^Department of Experimental Psychology, University of Oxford, Oxford OX2 6GG, United Kingdom, and; ^4^Oxford Centre for Human Brain Activity, University of Oxford, Oxford OX3 7JX, United Kingdom

**Keywords:** auditory processing, magnetoencephalography, multivariate decoding, rhythm processing, sensory prediction, temporal orienting

## Abstract

Temporal orienting improves sensory processing, akin to other top–down biases. However, it is unknown whether these improvements reflect increased neural gain to any stimuli presented at expected time points, or specific tuning to task-relevant stimulus aspects. Furthermore, while other top–down biases are selective, the extent of trade-offs across time is less well characterized. Here, we tested whether gain and/or tuning of auditory frequency processing in humans is modulated by rhythmic temporal expectations, and whether these modulations are specific to time points relevant for task performance. Healthy participants (*N* = 23) of either sex performed an auditory discrimination task while their brain activity was measured using magnetoencephalography/electroencephalography (M/EEG). Acoustic stimulation consisted of sequences of brief distractors interspersed with targets, presented in a rhythmic or jittered way. Target rhythmicity not only improved behavioral discrimination accuracy and M/EEG-based decoding of targets, but also of irrelevant distractors preceding these targets. To explain this finding in terms of increased sensitivity and/or sharpened tuning to auditory frequency, we estimated tuning curves based on M/EEG decoding results, with separate parameters describing gain and sharpness. The effect of rhythmic expectation on distractor decoding was linked to gain increase only, suggesting increased neural sensitivity to any stimuli presented at relevant time points.

**SIGNIFICANCE STATEMENT** Being able to predict when an event may happen can improve perception and action related to this event, likely due to the alignment of neural activity to the temporal structure of stimulus streams. However, it is unclear whether rhythmic increases in neural sensitivity are specific to task-relevant targets, and whether they competitively impair stimulus processing at unexpected time points. By combining magnetoencephalography and encephalographic recordings, neural decoding of auditory stimulus features, and modeling, we found that rhythmic expectation improved neural decoding of both relevant targets and irrelevant distractors presented and expected time points, but did not competitively impair stimulus processing at unexpected time points. Using a quantitative model, these results were linked to nonspecific neural gain increases due to rhythmic expectation.

## Abstract (precis)

Temporal orienting improves sensory processing, akin to other top–down biases. However, it is unknown whether these improvements reflect increased neural gain to any stimuli presented at expected time points, or specific tuning to task-relevant stimulus aspects. Furthermore, while other top–down biases are selective, the extent of trade-offs across time is less well characterized.

## Introduction

As our brains receive multiple sensory inputs over time, predicting when relevant events may happen can optimize perception and action (Nobre and van Ede, 2018). The behavioral and neural enhancement effects of temporal expectation are likely due to a time-specific increase in neural excitability coinciding with the expected target onset (Praamstra et al., 2006; Rohenkohl and Nobre, 2011; Zanto et al., 2011; Rohenkohl et al., 2012). In the context of rhythmic temporal expectation, these dynamic gain modulation effects have led to the hypothesis of neural entrainment, or phase alignment of ongoing neural activity to external rhythms. Invasive studies showed that attention to one of two rhythmic streams, presented in parallel, aligns the excitability peaks in primary cortical regions to the expected event onsets in the attended stream (Lakatos et al., 2008, 2013). Similar effects associated with neural entrainment have been observed in noninvasive human studies using electroencephalography (EEG) and magnetoencephalography (MEG; Stefanics et al., 2010; Cravo et al., 2013; Henry et al., 2014; Costa-Faidella et al., 2017; Ten Oever et al., 2017; but see Breska and Deouell, 2017).

However, it is unclear to what extent these rhythmic gain increases are target specific. First, it is unknown whether rhythmic expectations adaptively adjust gain due to temporal trade-offs, upregulating neural sensitivity to expected stimuli but competitively downregulating the neural processing of events occurring earlier or later. A recent behavioral study suggested that temporal cues enhance visual target processing at expected time points at the cost of unexpected time points (Denison et al., 2017), but whether rhythmic gain modulation operates in a similarly competitive manner, impairing neural processing at irrelevant phases of rhythmic stimulus streams relative to contexts in which no temporal expectation can be established, is an important open question, especially in light of a recently demonstrated double dissociation between temporal expectations based on rhythms versus specific intervals (Breska and Ivry, 2018).

Second, it is unclear whether rhythmic modulation of excitability is specific to relevant target features (akin to sharpened tuning of neural populations processing discriminant features), or nonspecific (i.e., also enhancing the processing of irrelevant distractors occurring in temporal proximity to targets, consistent with a true gain effect). Modeling of behavioral responses to visual targets presented under different kinds of attention has suggested that spatial and feature-based attention rely on gain and tuning mechanisms to a different extent (Ling et al., 2009). In the auditory modality, sustained attention to auditory rhythms (Lakatos et al., 2013; O'Connell et al., 2014) and gradually increasing temporal expectation (Jaramillo and Zador, 2011) sharpen frequency tuning (i.e., boost neural responses to the preferred acoustic frequency but dampen responses to other frequencies). However, it is unclear whether the same holds for rhythmic temporal orienting in more complex streams where distractors and targets cannot be easily separated by their frequency contents. In this case, both increased gain and sharpened tuning may provide plausible mechanisms of increasing sensory precision leading to improved processing of task-relevant features.

Time-specific modulation of sensory processing can be measured as changes in the quality of stimulus information encoded in neural signals. Multivariate decoding of electrophysiological data provides useful tools for quantifying the dynamic modulation of stimulus-related information (Garcia et al., 2013), as well as in the context of temporal expectation (Myers et al., 2015; van Ede et al., 2018). Here, we used multivariate decoding of MEG/EEG responses to examine how processing auditory targets (tone chords), and distractors (pure tones) presented at variable intervals, are modulated by rhythmic temporal expectation. The auditory modality was chosen as a natural testing ground for the mechanisms of neural alignment to rhythmic stimulus sequences (Obleser et al., 2017; Zoefel and VanRullen, 2017). We used a model of population tuning, with separate parameters coding for the gain and sharpness of auditory frequency decoding, and tested whether temporal expectation modulates the processing in a specific way (sharpening the tuning of frequencies useful for discriminating targets) or in a nonspecific way (adjusting the gain of all frequencies).

## Materials and Methods

### 

#### Participant sample

Healthy volunteers (*N* = 23, 12 female; mean age, 27.8 years; age range, 18–40 years) were invited to participate in the experiment upon written informed consent. All participants had normal hearing, no history of neurological or psychiatric diseases, and normal or corrected-to-normal vision. With the exception of one ambidextrous participant, all remaining participants were right handed by self-report. The experimental procedures were conducted in accordance with the Declaration of Helsinki (1991) and approved by the local ethics committee. One participant withdrew from the study before completing the experimental session, and their incomplete data were discarded from analysis, so that data from 22 participants were included in the analysis.

#### Experimental design and statistical analysis

##### Behavioral paradigm and stimulus design.

**Figure 1.**
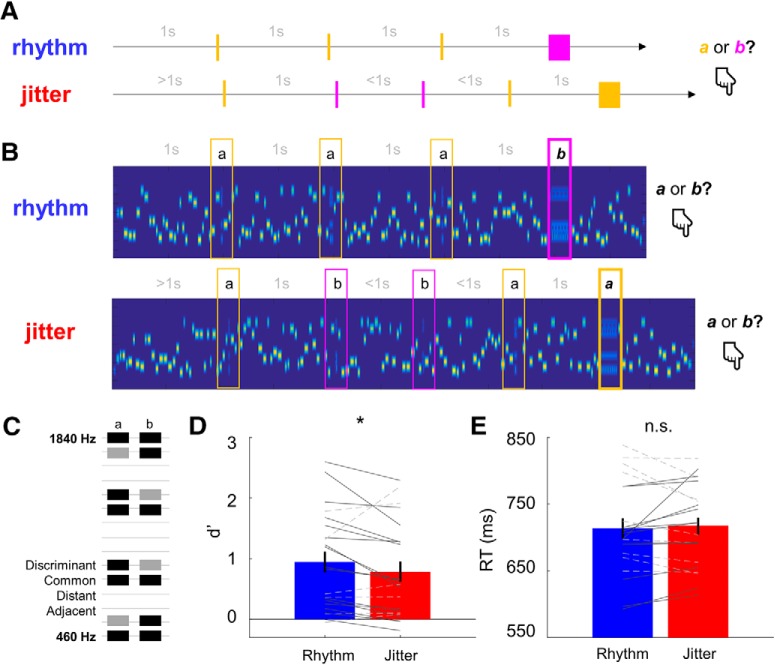
Behavioral paradigm and results. ***A***, Participants listened to sequences of pure tones interleaved with chords. For simplicity, only chords (but not pure tones) are shown on the time axes. A subset of these chords (20%) had a markedly longer duration and constituted targets. Upon hearing a target, participants were asked to categorize it as one of two predefined categories (“a” or “b”) using a button press. Sequences were presented in blocks of two experimental conditions, as follows: in the rhythmic condition, chords were presented with a fixed ISI of 1 s, and participants could form a temporal expectation of when to expect each upcoming chord. In the jittered condition, half of the ISIs, chosen at random were fixed at 1 s, and the remaining half ranged between 0.5 and 1.5 s, making chord onset unpredictable. ***B***, Spectrograms of example trials including pure tones surrounding the chords. ***C***, Chords were composed of eight pure tones each: four discriminant frequencies (two with a higher amplitude for each chord) and four common frequencies with equal amplitude for both chords. Pure tones were drawn from a larger set of 15 frequencies (range, 460–1840 Hz), including frequencies constituting the chords and other frequencies not included in the chords (adjacent to the discriminant frequencies or distant from them). ***D***, ***E***, Temporal expectation increased the participants' behavioral sensitivity (*d*′ values) in the chord discrimination task (marked by asterisk), but did not significantly affect their reaction times. Bars represent population means; solid (dashed) lines represent individual participants' data consistent (inconsistent) with the direction of the group effect; error bars denote the SEM. n.s., not significant.

Participants were instructed to listen to an acoustic stream comprising sequences of pure tones interleaved with chords (Fig. 1*A*,*B*). Each pure tone had a carrier frequency drawn randomly with replacement from a set of 15 logarithmically spaced frequencies spanning two octaves (range, 460–1840 Hz) and a duration drawn randomly with replacement from a set of five possible durations (23–43 ms in steps of 5 ms). The tones were tapered with a Hanning window (5 ms rise/fall time) and formed otherwise gapless sequences of spectrally and temporally nonoverlapping stimuli interspersed by chord stimuli.

Chords comprised 6 of the 15 frequencies used for the tones. Each chord was of one of two possible “types,” A, and B, depending on their spectral profile. Two of the six constituent tone amplitudes for the A and B tones were identical (“common”), while the remaining four amplitudes differed between A and B (“discriminant”: two with amplitudes higher for each chord; Fig. 1*C*, example). The six frequencies making up the chords were chosen pseudorandomly for each participant. Frequencies were chosen such that the chords could not be distinguished simply by overall pitch (i.e., the two discriminant frequencies with a larger amplitude in chord A were never both higher or lower than the other two discriminant frequencies). The remaining nine frequency bands that were not part of the chord could be divided into those “adjacent” to versus “distant” from the discriminant frequencies. The amplitude of each pure tone and chord was normalized by its mean loudness over time (Glasberg and Moore, 2002) to render the loudness of each stimulus in the sequence constant.

While most chord durations were drawn from the same set as for pure tones (23–43 ms), a subset of chords (20%) was markedly longer (165 ms) and constituted “targets.” The participants were instructed to listen for these target chords and to indicate quickly whenever they heard a long A or a B chord. In each trial, targets were presented after a sequence of pure tones interspersed with three to five short chords, and, upon hearing a longer chord, participants were asked to press one of two buttons (using their right index and middle fingers) assigned to chords A and B, respectively. Button assignment was counterbalanced across participants. Tone sequences continued for 715 ± 10 ms (mean ± SD) following target onset, including a 200 ms fadeout. The entire sequence duration ranged between 3.846 and 7.742 s with no difference in duration across conditions (mean ± SD; sequence duration, 5.683 ± 0.818 vs 5.676 ± 0.891 s in the rhythmic and jittered blocks, respectively). While performing the task, participants were instructed to maintain fixation on a centrally presented yellow fixation cross the color of which changed to green (red) following correct (incorrect) responses. Following feedback, a new trial started after 500 ± 100 ms (i.e., mean ± jitter) of fixation. In addition to the fixation cross, participants viewed silent grayscale videos of semistatic landscapes that were irrelevant to the task; these videos were displayed to prevent fatigue due to prolonged fixation on an otherwise empty screen. All visual stimulation was delivered using a projector (60 Hz refresh rate) in the experimenter room and transmitted to the MEG suite using a system of mirrors onto a screen located ∼90 cm from the participants.

In separate blocks, chords formed either a rhythmic sequence [with each two chords separated by a constant interstimulus interval (ISI) of 1 s] or a jittered sequence (with 50% of the ISIs equal to 1 s, 25% of the ISIs drawn randomly from 570 to 908 ms, and 25% drawn randomly from 1092 to 1430 ms). Each block contained 60 trials (targets) and 240 short chords. Our analysis focused completely on the chords preceded by an ISI of 1 s, so that any behavioral or neural differences between rhythmic and jittered blocks were not due to physical differences in stimuli presented immediately before a given chord. To obtain equal numbers of samples for the jittered and rhythmic conditions, each participant completed six blocks of target discrimination in jittered sequences and three blocks in rhythmic sequences. Block duration was kept constant across the two conditions. Block order was randomized per participant. Participants were not briefed on the ISI distribution between the rhythmic and jittered conditions.

Before performing the task, participants were familiarized with the stimuli. First, they heard 30 examples of each chord (A and B) in a randomized order, whereby A and B each contained two common frequencies and two discriminant frequencies at maximum amplitude. Next, they performed a training block of the chord discrimination task (using a jittered sequence) in which the relative amplitude of discriminant frequencies between chords A and B was adjusted (using a one up, two down staircase procedure with an adaptive step size) to ∼70% discrimination accuracy. Following the training session, task stimuli (including chords with individually adjusted amplitude of discriminant frequencies) were rendered off-line and stored as 16 bit .wav files at 48 kHz, delivered to the subjects' ears with tube ear phones and presented at a comfortable listening level (self-adjusted by each listener). The stimulus set was generated anew for each participant.

##### Neural data acquisition.

Each participant completed one session of concurrent EEG and MEG recording lasting ∼1 h for the entire experiment, excluding preparation. Participants were comfortably seated in the MEG scanner in a magnetically shielded room. MEG signals were acquired using a whole-head VectorView System (204 planar gradiometers, 102 magnetometers; Neuromag Oy, Elekta), sampled at a rate of 1 kHz and on-line bandpass filtered between 0.03 and 300 Hz. The participant's head position inside the scanner was continuously tracked using head position index coils placed at four distributed points on the scalp. Vertical electrooculogram (EOG) electrodes were placed above and below the right eye. Additionally, eye movements and pupil size were monitored using a remote infrared eye-tracker (sampling both eyes at 1 kHz and controlled via Psychophysics Toolbox; EyeLink 1000, SR Research; Cornelissen et al., 2002). Electrocardiogram (EKG) electrodes were placed on both wrists. EEG data were collected using 60 channels distributed across the scalp according to the international 10-10 positioning system at a sampling rate of 1 kHz.

##### Behavioral data analysis.

Behavioral responses to targets were analyzed with respect to their accuracy (percentage correct responses), sensitivity (*d*′), criterion, and reaction times (RTs). For each participant, trials with RTs longer than the individual median RT + 2 SDs were excluded from analysis. In the behavioral analyses, all responses were averaged in the rhythmic condition, while in the jittered condition only responses to targets preceded by an ISI of 1 s were taken into analysis to ensure that targets are preceded by the same ISI across conditions. Mean accuracy and RT data were subject to separate paired *t* tests and compared between the rhythmic and jittered conditions.

##### Neural data preprocessing.

The SPM12 toolbox (Wellcome Trust Centre for Neuroimaging, University College London, London, U.K.) for Matlab (MathWorks) was used to perform all preprocessing steps. Continuous M/EEG data were high-pass filtered at 0.1 Hz, notch filtered at 50 Hz and harmonics, and low-pass filtered at 200 Hz (all filters: fifth-order zero-phase Butterworth filters). Different channel types (EEG electrodes, MEG gradiometers, and magnetometers) were preprocessed together. Blink artifact correction was performed by detecting eye blink events in the vertical EOG channel and subtracting their two principal modes from the sensor data (Ille et al., 2002). Similarly, heart beats were detected in the EKG channel, and their two principal modes were subtracted from sensor data. EEG data (but not MEG data) were rereferenced to the average of all scalp channels.

##### Neural correlations with pure tone frequency.

To establish a basis for multivariate decoding of tone frequency from M/EEG data, we first tested whether M/EEG amplitude correlates with tone frequency in a mass-univariate way, and whether any such correlations can be source localized to auditory regions. Our rationale was that, given the short ISIs between the tones (∼33 ms), auditory frequency decoding would rest on the amplitude on relatively early-latency M/EEG signals likely arising from tonotopically organized regions (Su et al., 2014); consequently, different dipole orientations associated with neural activity evoked by different tone frequencies should translate into systematic variability in M/EEG amplitude. To test whether M/EEG amplitude covaries with pure tone frequency, we epoched M/EEG data from 200 ms before to 400 ms after each pure tone onset. The epochs were averaged for each tone frequency and smoothed with a 20 ms moving average window. The smoothing applied an effective low-pass frequency cutoff at ∼20 Hz, implemented to ensure that the time series of M/EEG activity evoked by each given tone are not dominated by sharp peaks of responses evoked by consecutive tones presented at ISI rates of ∼23–43 Hz. M/EEG time series smoothing has also been shown to improve subsequent decoding accuracy (Grootswagers et al., 2017). This resulted in 15 time series of mean M/EEG amplitude per participant and channel. Spearman's rank-order correlation coefficients were calculated per participant, channel, and time point between the mean M/EEG amplitude and tone frequency (specifically, with a monotonic vector in which the lowest frequency was assigned the lowest value and the highest frequency to the highest value). Spearman's rank-order correlation coefficients were chosen as they capture any monotonic relation between variables. To establish whether different M/EEG channel types (EEG electrodes, MEG magnetometers, and planar gradiometers) contain signals sensitive to the frequency of presented pure tones, the grand-average channel-by-time matrices of correlation coefficients between M/EEG amplitude and tone frequency were decomposed into principal modes using singular value decomposition. Per channel type, a set of principal modes (EEG: 7 modes of 60 original channels; magnetometers: 7 modes of 102 original channels; gradiometers: 11 modes of 204 original channels) explaining >95% of the original variance was retained. This form of principal component analysis-based data dimensionality reduction has been shown to substantially improve the accuracy of M/EEG multivariate decoding (Grootswagers et al., 2017), used in subsequent analysis steps (see below). The corresponding component weights were applied to individual participants' channel-by-time coefficient matrices and averaged. The resulting time series—effectively summarizing the individual participants' correlation time series across channels—were analyzed using cluster-based permutation tests (Maris and Oostenveld, 2007), which are an established method of analyzing M/EEG data, without making any assumptions about the normality of data distribution, while correcting for multiple comparison over time. Specifically, single-participant data were entered per channel type into separate cluster-based permutation one-sample *t* tests (which do not rely on any assumptions about the underlying data distribution), while correcting for multiple comparisons over time at a cluster-based threshold (*p* < 0.05).

**Figure 2.**
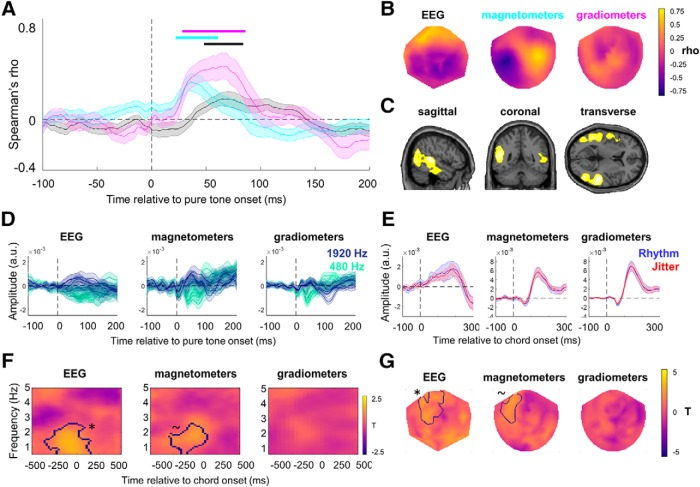
M/EEG sensor-level analysis. ***A***, Sensitivity to the amplitude of brief pure tones. Time series of correlations between M/EEG amplitudes and pure tone frequency (shaded areas, SEM) for different channel types (black, EEG; cyan, MEG magnetometers; magenta, MEG planar gradiometers). Each line represents the correlation coefficients between M/EEG amplitude and tone frequency, summarized across channels. Horizontal bars mark cluster-corrected (*p* < 0.05) significance against zero. ***B***, Topographies of the three respective correlation coefficients. ***C***, Orthogonal views of source estimates underlying the correlation peak, integrating across all channel types. Source reconstruction of the correlation coefficient time series (based on the multiple sparse priors algorithm; see Materials and Methods) were estimated using data fused across channel types, and were inferred in the contrast between the time window of the observed correlations (26–126 ms; chosen as a 100 ms time window around a peak correlation latency for all channel types) and the corresponding prestimulus baseline (126–26 ms before tone onset). All source estimates were significant at a threshold of *p* < 0.001 and correcting for multiple comparisons at a cluster level using an FWE-corrected *p* < 0.05. Slices centered at 52, −48, and 6 mm in MNI coordinates. ***D***, Grand averages (shaded areas, SEM) of summarized principal components of tone-evoked ERP/ERF amplitudes, per tone frequency (colored lines) and channel type (panels). ***E***, Grand averages (shaded areas, SEM) of summarized principal components of chord-evoked ERP/ERF amplitudes, per condition (blue, rhythmic, red, jittered) and channel type (panels). ***F***, PLV: effect of rhythms (time–frequency maps). Differences in PLV of M/EEG data at −500 to 500 ms relative to chord onset. Each panel shows the time–frequency map of mean *t* statistics averaged across channels for a given channel type. Contours outline the cluster of significant differences between rhythmic and jittered conditions, after correcting for multiple comparisons across channels and time–frequency points. Asterisk: cluster-based *p* < 0.05; tilde: cluster-based *p* < 0.1. ***G***, PLV: effect of rhythms (topographic maps). Each panel shows the topographical distribution of *t* statistic values at chord onset for the PLV estimate at 1 Hz. Contours are as described above.

While several other studies found monotonic effects on EEG amplitude (especially for frequencies >500 Hz) at both early (tens of milliseconds: Tabachnick and Toscano, 2018) and late (hundreds of milliseconds: Picton et al., 1978) latencies, nonmonotonic effects of tone frequency on EEG amplitude have also been reported (e.g., a quadratic relationship between tone frequency and N1 amplitude: Herrmann et al., 2013a). Although a visual inspection of our data suggested a primarily monotonic relationship between tone frequency and M/EEG amplitude (Fig. 2*D*), we have also tested for quadratic effects in the data. To this end, we have repeated the analysis described above, this time correlating M/EEG amplitudes with a vector representing the frequency axis quadratically (whereby the lowest and highest frequencies were assigned the highest value, and the medium frequency the lowest value). The remaining steps (principal component analysis and cluster-based permutation tests of correlation coefficient time series) were identical to that described above.

The time window in which we identified significant correlations between M/EEG amplitude and tone frequency was used for subsequent source reconstruction. Specifically, individual participants' channel-by-time correlation coefficient time series (for all channel types) were projected into source space using the multiple sparse priors algorithm under group constraints (Litvak and Friston, 2008), as implemented in SPM12; the group constraints ensure that responses are reconstructed in the same subset of sources for the entire participant sample. MEG and EEG data were source localized using a single generative model, which assumes that signals from different channel types arise from the same underlying current sources but map onto the sensors through different forward models (MEG, single shell; EEG, boundary element model), which also account for differences in units across data modalities (Henson et al., 2009). Source activity maps were smoothed in 3D with a Gaussian kernel at FWHM of 8 mm and tested for statistical significance in paired *t* tests between each participant's estimated sources [for the 26–126 ms time window (i.e., within a 100 ms time window around the correlation peak of 76 ms) for all channel types; see Results] and the corresponding prestimulus baseline. The reason for this time window selection was that, in source reconstruction using multiple sparse priors, it is usually recommended to include rise and fall times of signals peaking at a specific latency, since sources of activity are estimated based on signal variance across time rather than mere amplitude differences between channels at a specific time point (López et al., 2014). The resulting statistical parametric maps were thresholded at a peak-level uncorrected *p* value of <0.001 and corrected for multiple comparisons across voxels using a familywise error rate (FWE) of 0.05 under random field theory assumptions (Kilner et al., 2005). Sources were assigned probabilistic anatomical labels using a Neuromorphometrics atlas implemented in SPM12.

Finally, to plot tone-evoked and chord-evoked responses [event-related potentials (ERPs) and event-related fields (ERFs)] in the time domain, continuous M/EEG data were subject to singular value decomposition, as described above. Per participant, the principal components explaining >95% of the original variance were summarized and plotted over time in Figure 2, *D* and *E*.

##### Phase locking to rhythmic stimulus structure.

To test whether rhythmic presentation of chords influenced ongoing low-frequency activity, we quantified the phase-locking value (PLV; Lachaux et al., 1999) at chord onset. Since we were primarily interested in PLV at low frequencies including 1 Hz, we calculated instantaneous power and phase of ongoing activity in the 0.5–5 Hz range (in 0.1 Hz steps) at each time point from −500 to 500 ms (in 50 ms steps) relative to chord onset using a Morlet wavelet transform with a fixed time window of 2000 ms for each time–frequency estimate. To control for physical differences in stimulation between rhythmic and jittered blocks, we took into the analysis only these chords that were preceded and followed by an ISI of 1000 ms. By this criterion, the first chord was excluded in each trial, as any temporal expectation could only be established after its presentation. Based on the extracted phase values, per participant, channel, and condition (rhythmic vs jittered), we calculated PLV for each time–frequency point according to the following equation, where ϕ is a single-trial instantaneous phase of the wavelet transform, calculated for each of *N* trials, as follows:

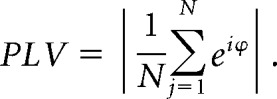
 Given that PLVs are bound between 0 and 1, we used the (paired, two-tailed) nonparametric test to assess whether phase locking is significantly different between the rhythmic and jittered conditions. To control for multiple comparisons across channels and time–frequency points, we used cluster-based permutation tests as implemented in Fieldtrip. The tests were conducted for each channel type (EEG electrodes, MEG magnetometers, and planar gradiometers) separately.

To ensure that the PLV analysis reveals effects that are not simply explained by differences in the amplitudes of ERPs/ERFs, we also extracted power estimates for each channel and time–frequency point and entered them into paired nonparametric cluster-based permutation tests, as described above.

##### Decoding pure tone frequency.

**Figure 3.**
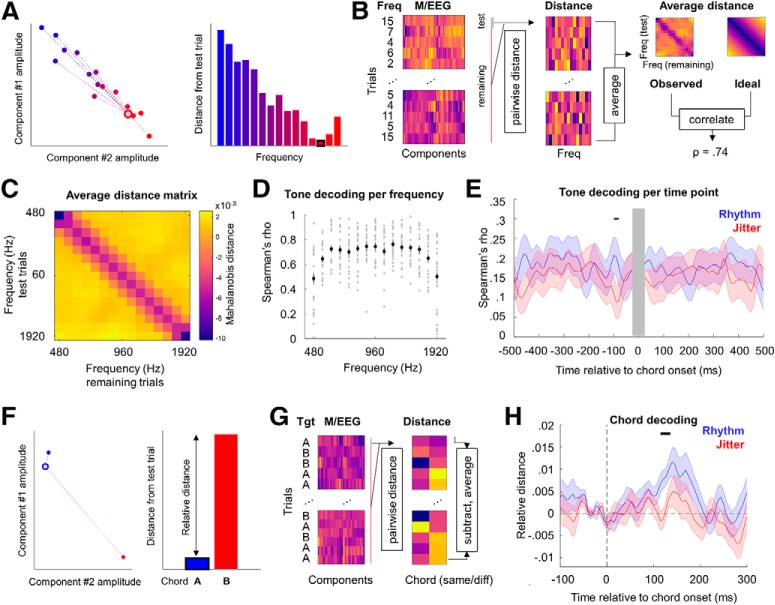
Decoding results. ***A***, Decoding methods were based on estimating multivariate Mahalanobis distance between M/EEG component amplitudes in a given (test) trial and average amplitudes calculated for all 15 frequencies, respectively (excluding the test trial). The left panel presents M/EEG component amplitudes for two example components (empty circle, test trial; solid circles, ERPs/ERFs calculated from the remaining trials; acoustic frequencies are color coded). Dashed lines on the left panel and bars on the right panel represent the multivariate distance between amplitudes observed in the test trial and the remaining trials. ***B***, Decoding methods as in ***A*** but for multiple components and multiple trials. The left panel presents M/EEG component amplitudes (in columns) per trial (in rows), with the tone identity (1–15) presented on each trial noted on the left. The middle panel presents the corresponding Mahalanobis distances per frequency (1–15, in columns) and trial (in rows). Each row consists of a vector of distances between the neural activity on the given trial and the average neural activity in response to each of the 15 frequencies (calculated from all other trials; i.e., the single-trial dissimilarity estimates between amplitudes measured for the tone frequency presented in a given trial and all other frequencies presented in the remaining trials). Frequency-tuning matrices (right), summarizing the population-level tuning curves, were obtained after averaging across trials, per frequency, resulting in a 15 × 15 similarity matrix between all tone frequencies (each row represents the distance of all test trials of a given frequency to the remaining trials sorted per frequency and is shown in columns). The observed frequency-tuning matrices (top right, example from one participant) were Spearman correlated with the “ideal” tuning matrix (bottom right), which consisted of the difference (in Hz) between pairs of tone frequencies. This correlation coefficient provided a summary statistic that reflects decoding quality (i.e., how closely the relative dissimilarity between tone-evoked neural responses; “observed” in the figure) corresponds to the relative dissimilarity between tone frequencies (“ideal” in the figure). ***C***, The observed grand average frequency-tuning matrix (averaged across participants, time points, and conditions). ***D***, Rank-order correlation coefficients between the estimated tuning and ideal tuning for each frequency (i.e., each row in the frequency-tuning matrix). Single grey dots mark single participants; black dots mark mean across participants. ***E***, Frequency decoding was significantly enhanced (cluster-corrected *p* < 0.05; black bar) in the rhythmic (blue) versus jittered (red) blocks between −100 and −80 ms before chord presentation. Gray box marks chord presentation latency, where no pure tones were presented and consequently no frequency decoding can be established. Shaded areas mark SEM across participants. ***F***, ***G***, Chord decoding was based on the same methods as in ***A*** and ***B***, except single-trial Mahalanobis distances were calculated for same versus different chords (instead of 15 different distractor frequencies). Only neural responses to short chords preceded by an ISI of 1 s were analyzed. ***H***, Chord decoding was significantly enhanced (cluster-corrected *p* < 0.05; black bar) in the rhythmic (blue) vs jittered (red) blocks between 115 and 136 ms following chord onset. Shaded areas mark the SEM across participants. Freq, Frequency.

To quantify population-level gain and tuning of neural responses to acoustic inputs, we used M/EEG-based decoding of pure tone frequency (Fig. 3*A*,*B*). The decoding methods are based on previous work in decoding continuous features (e.g., visual orientation) from M/EEG signals (Myers et al., 2015; Wolff et al., 2017; van Ede et al., 2018), and additional preprocessing steps are based on a recent study (Grootswagers et al., 2017) quantifying the effects of several analysis parameters on decoding accuracy, as detailed below; however, it should be noted that choices regarding optimal preprocessing and decoding methods are subject to an ongoing debate (Guggenmos et al., 2018; Kriegeskorte and Douglas, 2019). In this analysis, we calculated the trialwise Mahalanobis distances (De Maesschalck et al., 2000) of multivariate M/EEG signal amplitudes between the full range of pure tone frequencies and obtained frequency-by-frequency distance matrices, which were then parameterized in terms of gain and tuning (Ling et al., 2009). First, we segmented the M/EEG data from all channels (principal components; see above) into separate trials, defined in relation to pure tones presented from 500 ms before to 500 ms after each (short) chord. For instance, for tones presented 500 ms before a chord, we calculated (1) a vector of tone frequencies presented at this time point in each trial, and (2) a series of vectors of M/EEG amplitudes measured in the 26–126 ms time window (in steps of 5 ms) after this time point in each trial. The selected time window corresponded to the cluster in which a significant correlation between tone-evoked responses and tone frequency was observed (see Results). In a leave-one-out cross-validation approach (optimal for M/EEG decoding; Grootswagers et al., 2017), per trial, we calculated 15 pairwise distances between M/EEG amplitudes observed in a given test trial and mean vectors of M/EEG amplitudes averaged for each of the 15 tone frequencies in the remaining trials. The decision to perform our decoding analyses in a single-trial jack-knife approach is actually quite conservative, as calculating averages across a small number of trials during jack-knifing has been shown to further improve overall decoding (Grootswagers et al., 2017). The Mahalanobis distances were computed using the shrinkage-estimator covariance calculated from all trials excluding the test trial (Ledoit and Wolf, 2004). Although data from different channels (components) should in principle be orthogonal (given the previous dimensionality reduction using principal component analysis based on continuous data from the entire experiment) and therefore warrant calculating Euclidean rather than Mahalanobis distance values, trialwise data may still retain useful (noise) covariance that may improve decoding. Indeed, multivariate decoding based on Mahalanobis distance with Ledoit–Wolf shrinkage has been shown to outperform other correlation-based methods of measuring dissimilarity between brain states (Bobadilla-Suarez et al., 2019). Mahalanobis distance-based decoding has also been shown to be more reliable and less biased than linear classifiers and simple correlation-based metrics (Walther et al., 2016). Furthermore, rank correlation-based methods combined with data dimensionality reduction (e.g., in Mahalanobis distance calculation) have been shown to approach decoding accuracy achieved with linear discriminant analysis, Gaussian naive Bayes, and linear support vector machines (Grootswagers et al., 2017); thus, it is reasonable to assume that choosing Mahalanobis distance rather than rank correlation coefficient as a measure of neural dissimilarity further improves decoding accuracy, while at the same time being more computationally efficient than decoding based on other methods such as naive Bayes and support vector machines.

The minimum single-trial distance estimates observed in the 26–126 ms time window were selected to accommodate frequency-dependent peak latencies of the middle-latency auditory evoked potential (Woods et al., 1995). These distance estimates were then averaged across trials per tone frequency, resulting in a 15 × 15 distance matrix for all tones presented, at the relevant time bin relative to chord onset. This procedure was repeated for time bins relative to chord onset from 500 ms before to 500 ms after the chord, in steps of 10 ms. As before, only trials in which chords were preceded by an ISI of 1 s were taken into the analysis, which was conducted separately for rhythmic and jittered blocks. In this manner, we computed single-participant distance matrices for each time point relative to temporally predictable versus unpredictable chord presentation.

The quality of the decoding of pure tone frequency was assessed by comparing the estimated distance matrices with an “ideal decoding” distance matrix, with the lowest distance values along the diagonal and progressively higher distance values along the off-diagonal (Fig. 3*B*). To this end, for each participant and time point (from 500 ms before to 500 ms after the expected chord onset), we calculated the Spearman's rank correlation coefficient between the estimated distance matrix and the ideal distance matrix. Spearman's correlation coefficient was chosen to avoid making any assumptions about the shape of the ideal distance matrix (e.g., linear or log-spaced along the frequency axes), as it quantifies the strength of a monotonic relationship between two variables. The resulting time series of correlation coefficients were entered into a cluster-based permutation paired *t* test between rhythmic and jittered conditions. Time windows in which clusters of significant tests were observed were based on corrections for multiple comparisons over the entire time window (−500 to +500 ms) at a cluster-based threshold of *p* < 0.05 (two-tailed).

#### Neural data analysis

##### Decoding chords.

In addition to decoding pure tone frequency from the trial segments ranging from −500 to +500 ms relative to expected chord onset, we also decoded chord identity itself based on M/EEG data evoked by short chord presentation (Fig. 3*F*,*G*). The decoding methods were identical to those described above, except that instead of calculating pairwise distance values between a given trial and each of the 15 frequencies, we calculated pairwise distance values between a given (test) trial and (1) all remaining trials in which the same chord was presented as in the test trial as well as (2) all trials in which the other chord was presented. The relative distance was quantified per trial by subtracting the distance to “same chord” trials from the distance to “other chord” trials and averaged across trials. This procedure was repeated for each participant and time point from −100 to +400 ms relative to chord onset, separately for rhythmic and jittered conditions. Only chords preceded by an ISI 1 s were included in the analysis. The resulting single-subject time series of chord-decoding accuracy were subject to cluster-based permutation statistics, as described above.

##### Gain/tuning model of frequency encoding.

**Figure 4.**
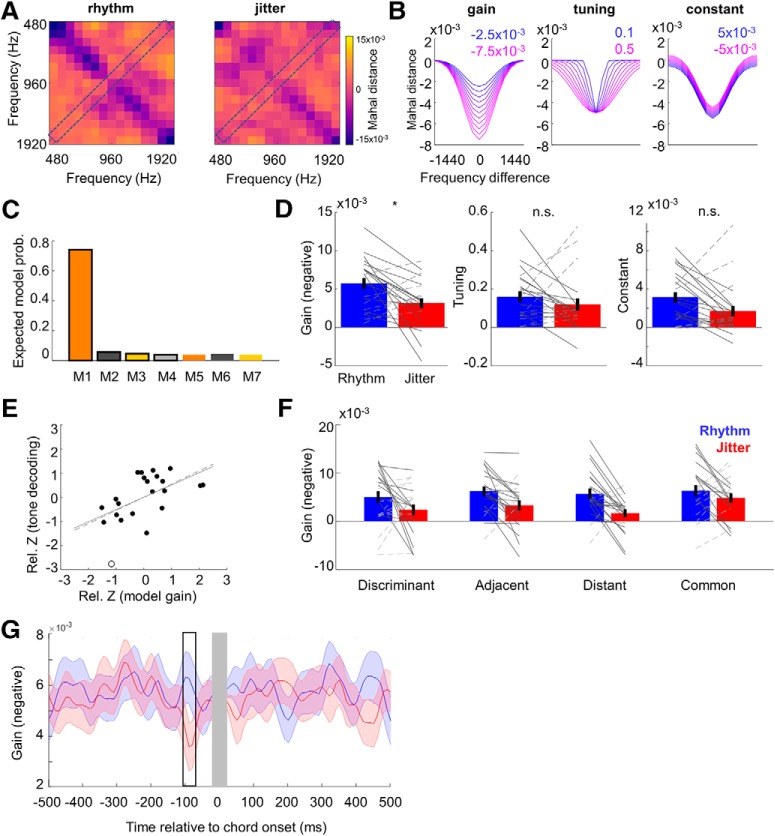
Modeling results. ***A***, Grand average frequency-tuning matrices for the rhythmic and jittered blocks, respectively (averaged between −100 and −80 ms before chord onset; Fig. 3*E*). Blue colors correspond to low distance (i.e., high similarity). ***B***, Effects of varying each of three free parameters in the gain/tuning model. The *x*-axis corresponds to the off-diagonal and the *y*-axis to the shading of a frequency-tuning matrix. ***C***, Model comparison of seven models (solid vs no outline, free vs fixed gain; orange vs gray, free vs fixed tuning; dark vs light, free vs fixed constant). The winning (full) model significantly outperformed the remaining models (see Results). ***D***, Effects of temporal expectation on model parameters. Only the gain parameter was significantly different (asterisk) between rhythmic and jittered contexts. ***E***, Correlation between the benefit in tone decoding (for rhythmic vs jittered blocks) and the difference in gain parameters (between rhythmic and jittered conditions) of the model. Dashed/solid line is the correlation coefficient slope before/after excluding an outlier (empty circle). ***F***, Relative gain (for rhythmic vs jittered conditions) did not significantly differ between models estimated separately for different frequency types (Fig. 1*C*). ***G***, The time course of the gain parameters for the entire analyzed time range (−500 to 500 ms relative to chord onset). Shaded areas mark SEMs. Blue, Rhythmic blocks; red, jittered blocks. Outline marks the latency of a significant effect reported in ***D***. Rel., Relative. n.s., not significant; *p < 0.05

To characterize the effects of rhythmic expectation on pure tone decoding in terms of gain and tuning to acoustic inputs, we fitted a simple model to individual participants' distance matrices, averaged across the time window in which significant results were observed (−100 to −80 ms before expected chord onset; see Results). Specifically, for each participant and condition, we fitted a three-parameter model to the observed distance matrices *Z*, with free parameters describing the gain *g* (i.e., M/EEG distance independent of relative tone frequency Δ*f*), tuning σ (i.e., a sharper or broader distribution of distance values along the relative tone frequency axis), and a constant term, *c* (i.e., mean distance across all relative tone frequencies), as follows:

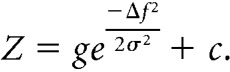
 This model equation is based on previous modeling work in humans investigating the gain and tuning effects of top–down attention in the visual domain (Ling et al., 2009). Figure 4*B* depicts the effects of each of these parameters on overall decoding matrices. Crucially, the gain parameter describes overall decoding quality (i.e., the relative similarity of neural responses to similar vs dissimilar frequencies, akin to nonspecific sensitivity modulation), while the tuning parameter describes the smoothness of the decoding matrix across the diagonal (i.e., the relative similarity of neural responses to identical vs adjacent frequencies, akin to frequency-specific sharpening). The resulting decoding matrices were assumed to be symmetric along the diagonal, based on previous literature suggesting overall frequency symmetry in spectrotemporal receptive fields of neurons in auditory cortex (Miller et al., 2002). All model fitting was performed using built-in Matlab robust fitting functions, with starting points based on the model fit to the grand average distance matrix (Fig. 3*C*). First, per participant, we fitted the full model with three free parameters, as well as a set of six reduced models in which each combination of the three parameters could be fixed to the value based on the model fit to the grand average distance matrix. In total, seven models were fitted for each participants' distance matrix (averaged across conditions). The models were compared using individual participants' Akaike information criterion (AIC) values, which reward models for their goodness of fit but penalize them for model complexity. The AIC values were treated as an approximation to log-model evidence and entered into a formal Bayesian model selection, as implemented in SPM12 (Peters et al., 2012). The winning model was then fitted per participant and condition, and the resulting parameter estimates were subject to three paired *t* tests (one per parameter) between fits to distance matrices estimated from rhythmic and jittered conditions. The *t* tests were corrected for multiple comparisons using a Bonferroni correction.

In addition to testing the effects of rhythm on gain and tuning across all tone frequencies, we also considered the possibility that gain and/or tuning modulation might be specific for those tone frequencies that were diagnostic of chord discrimination (Fig. 1*C*). To this end, we repeated the model-fitting procedure described above, this time fitting the (full) models separately to the following four categories of tone frequencies: (1) discriminant frequencies, whose amplitude differentiated between chords A and B; (2) frequencies adjacent to discriminant frequencies, which, however, do not constitute either chord A or B; (3) frequencies nonadjacent (distant) to discriminant frequencies, which do not belong to either chord A or B; and (4) frequencies common to chords A and B. The resulting parameter estimates were entered into a 2 × 4 repeated-measures ANOVA with factors temporal expectation (rhythmic vs jittered) and tone frequency (discriminant, adjacent, distant, common). We specifically tested for the interaction between the two factors, which would indicate that gain and/or tuning modulation by temporal expectation may depend on the type of tone frequency.

Finally, to test whether the effects of rhythm on tone (distractor) processing and chord (potential target) processing are interrelated, the following measures were contrasted between conditions (rhythmic vs jittered blocks), and the resulting differences were *z* scored and Pearson correlated across participants, as follows: (1) tone decoding (i.e., correlation coefficient with the ideal decoding matrix, averaged across the time window between −100 and −80 ms relative to chord onset); (2) the gain parameter of the gain/tuning model; (3) chord decoding (average Mahalanobis distance in the 115–136 ms postchord time window); and (4) behavioral accuracy. Correlations between measures were Bonferroni-corrected for multiple comparisons.

## Results

### Behavioral results

Behavioral performance in a chord discrimination task was affected by the temporal predictability of the chords (Fig. 1*D*,*E*). Participants discriminated the target chords more accurately in the rhythmic blocks than in the jittered blocks (mean ± SD: 72.04 ± 15.82% in the rhythmic blocks; 68.63 ± 16.07% in the jittered blocks; paired *t* test: *t*_(21)_ = 2.797, *p* = 0.011). This behavioral advantage was reflected in the participants' sensitivity *d*′ (mean ± SD: 0.944 ± 0.809 in the rhythmic blocks; 0.785 ± 0.782 in the jittered blocks; paired *t* test: *t*_(21)_ = 2.144, *p* = 0.044). There was no difference in criterion (mean ± SD: −0.008 ± 0.738 in the rhythmic blocks; 0.006 ± 0.756 in the jittered blocks; paired *t* test *t*_(21)_ = −0.222, *p* = 0.827). Mean reaction times also did not differ between rhythmic and jittered blocks (mean ± SD: 713 ± 69 ms in the rhythmic blocks; 717 ± 61 ms in the jittered blocks; paired *t* test: *t*_(21)_ = 0.751, *p* = 0.461), although the overall slow mean reaction times indicate that some participants did not follow the instructions to respond as soon as possible upon hearing the target chords, and instead waited until the end of the tone sequence.

### Activity in auditory regions covaries with tone frequency

To establish a basis for subsequent decoding, we tested whether tone frequency is reflected in evoked M/EEG signals. M/EEG amplitudes were correlated with tone frequency for all sensor types (Fig. 2*A*), as follows: for time series summarizing signal amplitudes obtained from MEG magnetometers (for details, see Materials and Methods), we observed a significant monotonic correlation between signal amplitude and tone frequency at 22–61 ms following tone onset (all *t*_(21)_ within the cluster >2.133; cluster-level *p* = 0.002); for MEG gradiometers, significant correlations were observed at 28–86 ms following tone onset (all *t*_(21)_ within the cluster >2.079; cluster-level *p* = 0.011); and finally, EEG amplitudes correlated with tone frequency at 48–83 ms following tone onset (all *t*_(21)_ within the cluster >2.087; cluster-level *p* = 0.028). Sensor topography of mean correlation coefficients are shown per channel type in Figure 2*B*. No significant clusters were observed for the analysis of quadratic effects of tone frequency on M/EEG amplitude (all clusters: *p* > 0.05).

Source reconstruction of the correlation coefficient time series, contrasting source-level activity estimates for the 100 ms time window around the latency (76 ms) at which the peak correlation between M/EEG amplitude and tone frequency has been observed (26–126 ms) and the corresponding prestimulus baseline (−126 to −26 ms relative to tone onset) revealed two significant clusters of source-level activity (Fig. 2*C*), encompassing bilateral primary auditory cortex (transverse temporal gyrus), planum temporale, and more lateral regions of superior temporal gyrus. MNI (Montreal Neurological Institute) coordinates of peak voxels, the corresponding statistics, and anatomical labels are reported in Table 1.

**Table 1.** Source reconstruction of the topography of correlation between M/EEG amplitudes and tone frequency

Cluster-level p_FWE-corr_	Number of voxels	Peak-level T	Peak-level Z	Peak MNI coordinates			Anatomical labels
				*x*	*y*	*z*	
0.005	4535	5.45	4.25	56	−38	6	Right MTG/STG
		5.42	4.24	46	−32	4	Right STG/MTG/PT/TTG
		5.06	4.05	48	−64	24	Right Ang/MOG/MTG
0.002	5215	5.15	4.10	−52	−44	30	Left SMG/PO/PT
		5.14	4.09	−52	−44	10	Left STG/MTG/PT
		5.03	4.03	−54	−26	24	Left PO/SMG/PoG/CO/PT
		4.93	3.97	−52	−10	12	Left TTG/CO

MTG, Middle temporal gyrus; STG, superior temporal gyrus; PT, planum temporale; TTG, transverse temporal gyrus (Heschl's gyrus); Ang, angular gyrus; MOG, middle occipital gyrus; SMG, supramarginal gyrus; PO, parietal operculum; PoG, postcentralgyrus; CO, central operculum.

### Rhythmic stimulus structure increases phase locking to chord onset

Rhythmic temporal expectation increased the low-frequency PLV at chord onset. Increased phase locking was observed in EEG channels (28 of 60 channels; paired *t* test statistic peaking at 1 Hz, −50 ms relative to chord onset; cluster-level *p* = 0.028). A similar trend was observed in MEG magnetometers (37 of 102 channels; paired *t* test statistic peaking at 1.8 Hz, 0 ms relative to chord onset; cluster-level *p* = 0.067; Fig. 2*F*,*G*), encompassing the chord presentation rate of 1 Hz. There was no accompanying increase in the power of ongoing activity for these or any other time–frequency points in the analyzed range (0.5–5 Hz, −1000 to 1000 ms relative to chord onset; all clusters *p* > 0.4), suggesting that the observed PLV increase is not merely due to power differences between conditions (van Diepen and Mazaheri, 2018). No significant differences in either PLV or power estimates were observed for MEG planar gradiometers (*p* > 0.1).

### Tone frequency can be decoded per time point relative to chord onset

Based on M/EEG amplitudes observed at all sensors, we calculated individual tone-by-tone Mahalanobis distance matrices, per time point, from −500 to +500 ms relative to chord onset (see Materials and Methods). Averaging across rhythmic and jittered blocks, the corresponding distance matrices showed significant above-chance tone frequency decoding for all inspected frequencies (Spearman's rank correlation coefficient ρ between the observed distance matrix and a matrix representing ideal decoding; one-sample *t* test: all *t*_(21)_ > 8.173, all *p* < 0.001; Fig. 3*D*) and time points (all *t*_(21)_ > 2.766, all *p* < 0.012). Crucially, tone decoding was also influenced by rhythmic temporal expectation (Fig. 3*E*). Specifically, when testing for differences between tone decoding per time point in rhythmic versus jittered blocks, a significant effect of temporal expectation was identified in a time window ranging between −100 and −80 ms before chord onset (permutation-based paired *t* test: all *t* > 2.136, cluster *p* = 0.016; Cohen's *d* = 0.900). In this time window, a higher correlation with the ideal decoding matrix was observed in rhythmic blocks (mean ± SD: ρ = 0.215 ± 0.173) than in the jittered blocks (mean ± SD: ρ = 0.070 ± 0.146).

### Chord decoding

In addition to establishing that pure tone frequency can be robustly decoded and identifying the effects of rhythmic expectation on processing tones presented at different latencies relative to chords, we also examined whether rhythmic expectation influences chord decoding itself. To this end, we calculated relative Mahalanobis distance between M/EEG topographies of responses evoked by chord presentation. A significant effect of rhythmic expectation was identified in the time window between 115 and 136 ms after chord onset (permutation-based paired *t* test between rhythmic and jittered blocks: all *t* > 2.099, cluster *p* = 0.044; Cohen's *d* = 0.783; Fig. 3*H*). In this time window, chord decoding was enhanced in the rhythmic condition (mean ± SD relative Mahalanobis distance = 0.009 ± 0.008), relative to the jittered condition (mean ± SD relative Mahalanobis distance = 0.001 ± 0.011).

### Temporal expectation modulates gain of population-level frequency processing

Having identified the significant effect of temporal expectation on pure tone decoding, we sought to investigate whether this effect on tone processing is due to gain and/or tuning sharpness modulation. To this end, we constructed and compared several alternative models of population-tuning curves that were fitted to the observed decoding matrices and parameterized them in terms of gain and tuning sharpness (Fig. 4*B*). First, we fitted a gain/tuning model with three free parameters (gain, tuning, constant)—as well as reduced models with different subsets of free parameters—to the observed decoding matrices (averaged across rhythmic and jittered blocks) in single participants. Bayesian model comparison using single-participant AIC values as an approximation to log model evidence (Peters et al., 2012) revealed that the full model outperformed the remaining models (Fig. 4*C*), with expected model probability given the data *p*(*m*|*y*) = 0.74 (all remaining models <0.05) and exceedance probability of >99.9% that the full model is better than any reduced model in describing the overall tone-decoding matrices (averaged across conditions).

Next, to test whether temporal expectation influences gain and/or tuning, we refitted the full model separately to decoding matrices obtained in each condition (rhythmic vs jittered blocks; Fig. 4*A*). A comparison of the obtained parameter estimates (Fig. 4*D*) revealed a significant effect of temporal expectation on the gain parameter (paired *t* test: *t*_(21)_ = −2.779, *p* = 0.011; Cohen's *d* = 0.783; please note that gain is expressed as a negative number, i.e., a more negative gain parameter corresponds to better decoding). This effect was specific to the time range for which significantly improved decoding was observed in the rhythmic conditions (i.e., −100 to −80 ms relative to chord onset; Fig. 4*G*). Furthermore, the median peak latency of the gain effect calculated for each participant was −80 ms relative to chord onset, coinciding with the latency of the group-level effect. Although the effect of experimental condition on the constant term was nominally significant, this test did not survive Bonferroni correction for multiple comparisons (*t*_(21)_ = 2.101, *p* = 0.048). The effect of rhythm on the tuning sharpness parameter was not significant (*t*_(21)_ = 1.039, *p* = 0.310).

Further, we tested whether the effect of temporal expectation on the gain parameter might be driven by a specific class of tone frequencies, such as those discriminating between the two chords that needed to be categorized by the participants. Thus, we repeated the model fitting for four classes of tones (discriminant, adjacent, distant, and common frequencies; for details, see Materials and Methods). A repeated-measures ANOVA revealed a main effect of temporal expectation, as identified above (*F*_(1,63)_ = 10.111, *p* = 0.004), but no main effect of frequency type (*F*_(3,63)_ = 2.253, *p* = 0.091) and, crucially, no interaction between the two (*F*_(3,63)_ = 1.725, *p* = 0.171). Therefore, the effect of temporal expectation on gain did not depend on tone type (Fig. 4*F*).

Finally, we investigated whether the neural and behavioral benefits of temporal expectation are correlated. Across participants, we correlated the *z*-scored differences between estimates of the following variables, obtained from the rhythmic and jittered condition, respectively, as follows: (1) gain parameter; (2) tone decoding (i.e., rank-order correlation with the ideal decoding matrix); (3) chord decoding (Mahalanobis distance); and (4) behavioral accuracy. A significant correlation was observed between the effect of temporal expectation on the gain parameter and the underlying tone-decoding modulation by temporal expectation (*r* = −0.549, *p* = 0.008; significant after Bonferroni correction for multiple comparisons across pairs of variables; Fig. 4*E*). After removing one outlier participant whose data were characterized by the Cook's distance metric exceeding the mean, the correlation remained significant (*r* = −0.511, *p* = 0.018). No other correlations were found to be significant.

## Discussion

We have shown that rhythmic temporal expectation improves target chord discrimination accuracy, increases the phase locking of neural signals at chord onset, as well as improves M/EEG-based chord decoding. Interestingly, we also show that before chord (i.e., a potential target) onset, temporal expectation improves the decoding of irrelevant distractors (pure tones). This beneficial effect can be modeled as increased gain to any stimuli (auditory frequencies) presented at time points adjacent to expected chord onset, and independent of whether processing these frequencies may be beneficial for chord discrimination.

In the present study, rhythm-induced temporal expectation increased the participants' sensitivity to target chords. Similar behavioral improvements have been reported previously, typically accompanied by shorter RTs to expected targets (Jaramillo and Zador, 2011; Rimmele et al., 2011; Rohenkohl et al., 2012; Cravo et al., 2013). While here we only compared responses to stimuli presented in rhythmic (isochronous) and jittered sequences while controlling for physical differences between conditions (i.e., only selecting targets preceded by identical intervals), other researchers have also found accuracy improvements in quasirhythmic sequences when acoustic targets were presented following a mean interval versus at other intervals (but see Jones, 2015; Herrmann et al., 2016). Another recent study has shown that, while different types of temporal expectation might lead to accuracy benefits, rhythmic expectation specifically shortens RTs (Morillon et al., 2016). However, RTs have been suggested to be more sensitive to temporal orienting in detection tasks than in discrimination tasks (Correa et al., 2004). While in the present study participants were instructed to discriminate chords by responding as soon as possible after hearing a target, auditory streams continued for several hundreds of milliseconds following target offset, which may have resulted in overall slow responses (Fig. 1*E*) and a reduced sensitivity to detect RT effects.

Beyond the increased behavioral sensitivity to target chords, we also observed improved neural decoding of short chords in the rhythmic versus jittered condition. Previous auditory studies have shown that rhythmic presentation of targets presented at a low signal-to-noise ratio amid continuous distractors increases their detectability (Lawrance et al., 2014; Rajendran et al., 2016). Similar findings in visual studies (Ten Oever et al., 2017) have been linked to increased phase locking of neural activity around the expected target onset. In our study, rhythm-induced temporal expectation increased phase locking of M/EEG signals at the chord presentation rate (but not chord-evoked ERF/ERP amplitude), which is consistent with previous reports (Cravo et al., 2013; Henry et al., 2014; Costa-Faidella et al., 2017) and with the entrainment hypothesis (Schroeder and Lakatos, 2009; for review, see Haegens and Zion Golumbic, 2018), which posits that external rhythms synchronize low-frequency neural activity and create time windows of increased sensitivity to stimuli presented at expected latencies. However, since phase locking has been shown to also increase due to interval-based expectations (Breska and Deouell, 2014), it may not be a specific measure of rhythm-induced temporal expectation.

In addition to improving the decoding of short chords (potential targets), rhythmic expectation also improved the decoding of pure tones (irrelevant distractors) preceding the chords. Current hypotheses are largely agnostic to whether neural alignment to external rhythms also results in temporal trade-offs, creating windows of decreased sensitivity at unexpected or irrelevant latencies. Such competitive effects have been described in the domain of spatial visual attention (Carrasco, 2011); however, temporal expectations have been suggested to play a largely modulatory role, amplifying the influence of other (e.g., spatial) sources of top–down control rather than themselves exerting strong influences on neural processing (Rohenkohl et al., 2014). While processing limitations over time have long been established (e.g., in the attentional blink literature; Shapiro et al., 1994), temporal expectations can in fact prevent attentional blink: knowing when subsequent targets will occur can improve their processing and diminish the (detrimental) effects of preceding targets (Martens and Johnson, 2005). Similarly, cues predicting target latency do seem not only to improve target processing but also to impair processing targets that appear at invalidly cued latencies (Denison et al., 2017). In this study, however, we did not observe impaired processing of stimuli presented at unexpected time points, which would likely manifest as impaired decoding and lower gain in the rhythmic versus jittered condition several hundred milliseconds before and after chord onset. Instead, our results suggest that while rhythmic auditory expectation increases sensitivity at expected latencies, it does not necessarily involve temporal trade-off with unexpected latencies.

We also considered another possible trade-off, namely temporal expectation boosting the processing of relevant targets at the expense of irrelevant distractors. A recent EEG study showed that anticipatory cues not only boost visual target decoding, but also decrease its interference by distractors presented just after the targets, possibly reflecting a protective time window for target processing (van Ede et al., 2018). However, as shown in other contexts (Rohenkohl et al., 2011; Morillon et al., 2016; Breska and Ivry, 2018), rhythm-induced expectations may not operate in the same manner as cue-induced expectations. Indeed, rhythms can facilitate performance independently of whether they are predictive of when the relevant targets may appear (Sanabria et al., 2011). In some cases, performance is superior for those targets that occur on-beat, even if targets more often occur off-beat (Breska and Deouell, 2014). In line with the latter results, our findings show improved decoding of irrelevant stimuli if they are presented at latencies leading up to the expected onsets of potential targets. While these differences were observed between −100 and −80 ms but not at even shorter latencies before chord onset, it is worth noting that decoding pure tones was based on M/EEG activity evoked by these tones (i.e., with a lag of up to 126 ms). Thus, just before chord onset, interference between chord-evoked activity and tone-evoked activity may have compromised tone decoding. Given the previously observed differences between rhythm- and cue-induced temporal expectations, it remains an important open question whether the type of temporal expectation manipulations, and/or individual participants' strategies in generating these expectations, may influence the latencies at which improved decoding can be observed.

To interpret the finding that rhythm-based expectation improves the decoding of irrelevant distractors before the expected target onset, we used a model that independently parameterized the gain and tuning of population-level frequency coding and found that rhythm-based expectation increased the gain of pure tone decoding. No evidence was found for the sharpening of tuning induced by temporal expectation. This suggests that, unlike in previous (animal) studies showing that sustained attention to acoustic rhythms (O'Connell et al., 2014) or increased target onset probability (Jaramillo and Zador, 2011) sharpen frequency tuning, in the current study rhythm-induced expectations—at the level of population-based decoding—could be linked to dynamic modulations of gain, more akin to classical neuromodulatory effects (Auksztulewicz et al., 2018). Previous behavioral modeling studies showed that rhythm-based expectation does indeed increase the signal-to-noise gain of sensory evidence in a visual discrimination task (Rohenkohl et al., 2012). Here, the gain effect was independent of whether the specific frequencies were useful for discriminating potential targets, further supporting the notion that the rhythmic increases of sensitivity are independent of stimulus relevance (Breska and Deouell, 2014). It is worth noting that, unlike in the previous electrophysiology studies (Lakatos et al., 2013; O'Connell et al., 2014), the perceptual discriminations here were based on chords with no overall frequency differences, showing that rhythm-induced expectations can work on composite representations. It remains to be tested whether the rhythm-induced dynamic gain modulation generalizes across data modalities and species (e.g., invasive recordings in animal models).

On a methodological note, our study is the first to show robust M/EEG-based multivariate decoding of pure tone frequency across a broad range of frequencies. While recent studies have brought substantial advances in decoding auditory features, studies using discreet stimuli have focused on decoding complex features such as pitch/rate modulation based on spectral information in MEG signals (Herrmann et al., 2013b) or bistable percepts based on evoked MEG responses (Billig et al., 2018). In the domain of speech decoding, speech-evoked responses can be used to decode vowel categories (Yi et al., 2017), but typically a combination of complex spectral features is used to decode the speech envelope (Luo and Poeppel, 2007; Ng et al., 2013; de Cheveigné et al., 2018). Here, robust decoding of pure tone frequency was achieved based on relatively early M/EEG response latencies (<100 ms) evoked by very brief tones (∼33 ms), despite their presentation in gapless streams. Finally, topographies of correlations between M/EEG amplitudes and tone frequency could be localized to auditory regions, suggesting that frequency decoding is based on sensory processing of acoustic features rather than on hierarchically higher activity related to complex percepts.

In summary, we have demonstrated that rhythmic expectation enhances population responses not only to task-relevant targets, but also to task-irrelevant distractors preceding potential targets. The latter effect could be explained in terms of nonspecific neural gain changes at time points adjacent to rhythm-induced expectation of relevant latencies. These findings speak against necessary temporal trade-offs in rhythmic orienting and support theories of neural alignment to the rhythmic structure of stimulus streams, plausibly mediated by dynamic neuromodulation.

## References

[B1] Auksztulewicz R, Schwiedrzik CM, Thesen T, Doyle W, Devinsky O, Nobre AC, Schroeder CE, Friston KJ, Melloni L (2018) Not all predictions are equal: “what” and “when” predictions modulate activity in auditory cortex through different mechanisms. J Neurosci 38:8680–8693. 10.1523/JNEUROSCI.0369-18.2018 30143578 PMC6170983

[B2] Billig AJ, Davis MH, Carlyon RP (2018) Neural decoding of bistable sounds reveals an effect of intention on perceptual organization. J Neurosci 38:2844–2853. 10.1523/JNEUROSCI.3022-17.2018 29440556 PMC5852662

[B3] Bobadilla-Suarez S, Ahlheim C, Mehrotra A, Panos A, Love BC (2019) Measures of neural similarity. BioRxiv. Advance online publication. Retrieved October 27, 2019. doi:10.1101/439893 10.1101/439893 PMC7671987 33225218

[B4] Breska A, Deouell LY (2014) Automatic bias of temporal expectations following temporally regular input independently of high-level temporal expectation. J Cogn Neurosci 26:1555–1571. 10.1162/jocn_a_00564 24392898

[B5] Breska A, Deouell LY (2017) Neural mechanisms of rhythm-based temporal prediction: delta phase-locking reflects temporal predictability but not rhythmic entrainment. PLoS Biol 15:e2001665. 10.1371/journal.pbio.2001665 28187128 PMC5302287

[B6] Breska A, Ivry RB (2018) Double dissociation of single-interval and rhythmic temporal prediction in cerebellar degeneration and Parkinson's disease. Proc Natl Acad Sci U S A 115:12283–12288. 10.1073/pnas.1810596115 30425170 PMC6275527

[B7] Carrasco M (2011) Visual attention: the past 25 years. Vision Res 51:1484–1525. 10.1016/j.visres.2011.04.012 21549742 PMC3390154

[B8] Cornelissen FW, Peters EM, Palmer J (2002) The eyelink toolbox: eye tracking with MATLAB and the psychophysics toolbox. Behav Res Methods Instrum Comput 34:613–617. 10.3758/BF03195489 12564564

[B9] Correa A, Lupiáñez J, Milliken B, Tudela P (2004) Endogenous temporal orienting of attention in detection and discrimination tasks. Percept Psychophys 66:264–278. 10.3758/BF03194878 15129748

[B10] Costa-Faidella J, Sussman ES, Escera C (2017) Selective entrainment of brain oscillations drives auditory perceptual organization. Neuroimage 159:195–206. 10.1016/j.neuroimage.2017.07.056 28757195 PMC5671350

[B11] Cravo AM, Rohenkohl G, Wyart V, Nobre AC (2013) Temporal expectation enhances contrast sensitivity by phase entrainment of low-frequency oscillations in visual cortex. J Neurosci 33:4002–4010. 10.1523/JNEUROSCI.4675-12.2013 23447609 PMC3638366

[B12] de Cheveigné A, Wong DDE, Di Liberto GM, Hjortkjær J, Slaney M, Lalor E (2018) Decoding the auditory brain with canonical component analysis. Neuroimage 172:206–216. 10.1016/j.neuroimage.2018.01.033 29378317

[B13] De Maesschalck R, Jouan-Rimbaud D, Massart DL (2000) The mahalanobis distance. Chemom Intell Lab Syst 50:1–18. 10.1016/S0169-7439(99)00047-7

[B14] Denison RN, Heeger DJ, Carrasco M (2017) Attention flexibly trades off across points in time. Psychon Bull Rev 24:1142–1151. 10.3758/s13423-016-1216-1 28054311 PMC5496802

[B15] Garcia JO, Srinivasan R, Serences JT (2013) Near-real-time feature-selective modulations in human cortex. Curr Biol 23:515–522. 10.1016/j.cub.2013.02.013 23477721 PMC3608396

[B16] Glasberg BR, Moore BCJ (2002) A model of loudness applicable to time-varying sounds. J Audio Eng Soc 50:331–342.

[B17] Grootswagers T, Wardle SG, Carlson TA (2017) Decoding dynamic brain patterns from evoked responses: a tutorial on multivariate pattern analysis applied to time series neuroimaging data. J Cogn Neurosci 29:677–697. 10.1162/jocn_a_01068 27779910

[B18] Guggenmos M, Sterzer P, Cichy RM (2018) Multivariate pattern analysis for MEG: a comparison of dissimilarity measures. Neuroimage 173:434–447. 10.1016/j.neuroimage.2018.02.044 29499313

[B19] Haegens S, Zion Golumbic E (2018) Rhythmic facilitation of sensory processing: a critical review. Neurosci Biobehav Rev 86:150–165. 10.1016/j.neubiorev.2017.12.002 29223770

[B20] Henry MJ, Herrmann B, Obleser J (2014) Entrained neural oscillations in multiple frequency bands comodulate behavior. Proc Natl Acad Sci U S A 111:14935–14940. 10.1073/pnas.1408741111 25267634 PMC4205645

[B21] Henson RN, Mouchlianitis E, Friston KJ (2009) MEG and EEG data fusion: simultaneous localisation o face-evoked responses. Neuroimage 47:581–589. 10.1016/j.neuroimage.2009.04.063 19398023 PMC2912501

[B22] Herrmann B, Henry MJ, Obleser J (2013a) Frequency-specific adaptation in human auditory cortex depends on the spectral variance in the acoustic stimulation. J Neurophysiol 109:2086–2096. 10.1152/jn.00907.2012 23343904

[B23] Herrmann B, Henry MJ, Grigutsch M, Obleser J (2013b) Oscillatory phase dynamics in neural entrainment underpin illusory percepts of time. J Neurosci 33:15799–15809. 10.1523/JNEUROSCI.1434-13.2013 24089487 PMC6618472

[B24] Herrmann B, Henry MJ, Haegens S, Obleser J (2016) Temporal expectations and neural amplitude fluctuations in auditory cortex interactively influence perception. Neuroimage 124:487–497. 10.1016/j.neuroimage.2015.09.019 26386347

[B25] Ille N, Berg P, Scherg M (2002) Artifact correction of the ongoing EEG using spatial filters based on artefact and brain signal topographies. J Clin Neurophysiol 19:113–124. 10.1097/00004691-200203000-00002 11997722

[B26] Jaramillo S, Zador AM (2011) The auditory cortex mediates the perceptual effects of acoustic temporal expectation. Nat Neurosci 14:246–251. 10.1038/nn.2688 21170056 PMC3152437

[B27] Jones A (2015) Independent effects of bottom-up temporal expectancy and top-down spatial attention. an audiovisual study using rhythmic cueing. Front Integr Neurosci 8:96. 10.3389/fnint.2014.00096 25610378 PMC4285055

[B28] Kilner JM, Kiebel SJ, Friston KJ (2005) Applications of random field theory to electrophysiology. Neurosci Lett 374:174–178. 10.1016/j.neulet.2004.10.052 15663957

[B29] Kriegeskorte N, Douglas PK (2019) Interpreting encoding and decoding models. Curr Opin Neurobiol 55:167–179. 10.1016/j.conb.2019.04.002 31039527 PMC6705607

[B30] Lachaux JP, Rodriguez E, Martinerie J, Varela FJ (1999) Measuring phase synchrony in brain signals. Hum Brain Mapp 8:194–208. 10.1002/(SICI)1097-0193(1999)8:4<194::AID-HBM4>3.0.CO;2-C 10619414 PMC6873296

[B31] Lakatos P, Karmos G, Mehta AD, Ulbert I, Schroeder CE (2008) Entrainment of neuronal oscillations as a mechanism of attentional selection. Science 320:110–113. 10.1126/science.1154735 18388295

[B32] Lakatos P, Musacchia G, O'Connel MN, Falchier AY, Javitt DC, Schroeder CE (2013) The spectrotemporal filter mechanism of auditory selective attention. Neuron 77:750–761. 10.1016/j.neuron.2012.11.034 23439126 PMC3583016

[B33] Lawrance EL, Harper NS, Cooke JE, Schnupp JW (2014) Temporal predictability enhances auditory detection. J Acoust Soc Am 135:EL357–EL363. 10.1121/1.4879667 24907846 PMC4491983

[B34] Ledoit O, Wolf M (2004) Honey, I shrunk the sample covariance matrix. J Portf Manag 30:110–119. 10.3905/jpm.2004.110

[B35] Ling S, Liu T, Carrasco M (2009) How spatial and feature-based attention affect the gain and tuning of population responses. Vision Res 49:1194–1204. 10.1016/j.visres.2008.05.025 18590754 PMC2696585

[B36] Litvak V, Friston K (2008) Electromagnetic source reconstruction for group studies. Neuroimage 42:1490–1498. 10.1016/j.neuroimage.2008.06.022 18639641 PMC2581487

[B37] López JD, Litvak V, Espinosa JJ, Friston K, Barnes GR (2014) Algorithmic procedures for bayesian MEG/EEG source reconstruction in SPM. Neuroimage 84:476–487. 10.1016/j.neuroimage.2013.09.002 24041874 PMC3913905

[B38] Luo H, Poeppel D (2007) Phase patterns of neuronal responses reliably discriminate speech in human auditory cortex. Neuron 54:1001–1010. 10.1016/j.neuron.2007.06.004 17582338 PMC2703451

[B39] Maris E, Oostenveld R (2007) Nonparametric statistical testing of EEG- and MEG-data. J Neurosci Methods 164:177–190. 10.1016/j.jneumeth.2007.03.024 17517438

[B40] Martens S, Johnson A (2005) Timing attention: cuing target onset interval attenuates the attentional blink. Mem Cognit 33:234–240. 10.3758/BF03195312 16028578

[B41] Miller LM, Escabí MA, Read HL, Schreiner CE (2002) Spectrotemporal receptive fields in the lemniscal auditory thalamus and cortex. J Neurophysiol 87:516–527. 10.1152/jn.00395.2001 11784767

[B42] Morillon B, Schroeder CE, Wyart V, Arnal LH (2016) Temporal prediction in lieu of periodic stimulation. J Neurosci 36:2342–2347. 10.1523/JNEUROSCI.0836-15.2016 26911682 PMC4860448

[B43] Myers NE, Rohenkohl G, Wyart V, Woolrich MW, Nobre AC, Stokes MG (2015) Testing sensory evidence against mnemonic templates. Elife 4:e09000. 10.7554/eLife.09000 26653854 PMC4755744

[B44] Ng BS, Logothetis NK, Kayser C (2013) EEG phase patterns reflect the selectivity of neural firing. Cereb Cortex 23:389–398. 10.1093/cercor/bhs031 22345353

[B45] Nobre AC, van Ede F (2018) Anticipated moments: temporal structure in attention. Nat Rev Neurosci 19:34–48. 10.1038/nrn.2017.141 29213134

[B46] Obleser J, Henry MJ, Lakatos P (2017) What do we talk about when we talk about rhythm? PLoS Biol 15:e1002615. 10.1371/journal.pbio.1002615 28926570 PMC5604933

[B47] O'Connell MN, Barczak A, Schroeder CE, Lakatos P (2014) Layer specific sharpening of frequency tuning by selective attention in primary auditory cortex. J Neurosci 34:16496–16508. 10.1523/JNEUROSCI.2055-14.2014 25471586 PMC4252556

[B48] Peters J, Miedl SF, Büchel C (2012) Formal comparison of dual-parameter temporal discounting models in controls and pathological gamblers. PLoS One 7:e47225. 10.1371/journal.pone.0047225 23226198 PMC3511467

[B49] Picton TW, Woods DL, Proulx GB (1978) Human auditory sustained potentials. II. stimulus relationships. Electroencephalogr Clin Neurophysiol 45:198–210. 10.1016/0013-4694(78)90004-4 78830

[B50] Praamstra P, Kourtis D, Kwok HF, Oostenveld R (2006) Neurophysiology of implicit timing in serial choice reaction-time performance. J Neurosci 26:5448–5455. 10.1523/JNEUROSCI.0440-06.2006 16707797 PMC6675318

[B51] Rajendran VG, Harper NS, Abdel-Latif KH, Schnupp JW (2016) Rhythm facilitates the detection of repeating sound patterns. Front Neurosci 10:9. 10.3389/fnins.2016.00009 26858589 PMC4731741

[B52] Rimmele J, Jolsvai H, Sussman E (2011) Auditory target detection is affected by implicit temporal and spatial expectations. J Cogn Neurosci 23:1136–1147. 10.1162/jocn.2010.21437 20146603 PMC2894284

[B53] Rohenkohl G, Nobre AC (2011) α oscillations related to anticipatory attention follow temporal expectations. J Neurosci 31:14076–14084. 10.1523/JNEUROSCI.3387-11.2011 21976492 PMC4235253

[B54] Rohenkohl G, Coull JT, Nobre AC (2011) Behavioural dissociation between exogenous and endogenous temporal orienting of attention. PLoS One 6:e14620. 10.1371/journal.pone.0014620 21297968 PMC3030556

[B55] Rohenkohl G, Cravo AM, Wyart V, Nobre AC (2012) Temporal expectation improves the quality of sensory information. J Neurosci 32:8424–8428. 10.1523/JNEUROSCI.0804-12.2012 22699922 PMC4235252

[B56] Rohenkohl G, Gould IC, Pessoa J, Nobre AC (2014) Combining spatial and temporal expectations to improve visual perception. J Vis 14:8. 10.1167/14.4.8 24722562 PMC3983934

[B57] Sanabria D, Capizzi M, Correa A (2011) Rhythms that speed you up. J Exp Psychol Hum Percept Perform 37:236–244. 10.1037/a0019956 20718571

[B58] Schroeder CE, Lakatos P (2009) Low-frequency neuronal oscillations as instruments of sensory selection. Trends Neurosci 32:9–18. 10.1016/j.tins.2008.09.012 19012975 PMC2990947

[B59] Shapiro KL, Raymond JE, Arnell KM (1994) Attention to visual pattern information produces the attentional blink in rapid serial visual presentation. J Exp Psychol Hum Percept Perform 20:357–371. 10.1037/0096-1523.20.2.357 8189198

[B60] Stefanics G, Hangya B, Hernádi I, Winkler I, Lakatos P, Ulbert I (2010) Phase entrainment of human delta oscillations can mediate the effects of expectation on reaction speed. J Neurosci 30:13578–13585. 10.1523/JNEUROSCI.0703-10.2010 20943899 PMC4427664

[B61] Su L, Zulfiqar I, Jamshed F, Fonteneau E, Marslen-Wilson W (2014) Mapping tonotopic organization in human temporal cortex: representational similarity analysis in EMEG source space. Front Neurosci 8:368. 10.3389/fnins.2014.00368 25429257 PMC4228977

[B62] Tabachnick AR, Toscano JC (2018) Perceptual encoding in auditory brainstem responses: effects of stimulus frequency. J Speech Lang Hear Res 61:2364–2375. 10.1044/2018_JSLHR-H-17-0486 30193361

[B63] Ten Oever S, Schroeder CE, Poeppel D, van Atteveldt N, Mehta AD, Mégevand P, Groppe DM, Zion-Golumbic E (2017) Low-frequency cortical oscillations entrain to subthreshold rhythmic auditory stimuli. J Neurosci 37:4903–4912. 10.1523/JNEUROSCI.3658-16.2017 28411273 PMC5426181

[B64] van Diepen RM, Mazaheri A (2018) The caveats of observing inter-trial phase-coherence in cognitive neuroscience. Sci Rep 8:2990. 10.1038/s41598-018-20423-z 29445210 PMC5813180

[B65] van Ede F, Chekroud SR, Stokes MG, Nobre AC (2018) Decoding the influence of anticipatory states on visual perception in the presence of temporal distractors. Nat Commun 9:1449. 10.1038/s41467-018-03960-z 29654312 PMC5899132

[B66] Walther A, Nili H, Ejaz N, Alink A, Kriegeskorte N, Diedrichsen J (2016) Reliability of dissimilarity measures for multi-voxel pattern analysis. Neuroimage 137:188–200. 10.1016/j.neuroimage.2015.12.012 26707889

[B67] Wolff MJ, Jochim J, Akyürek EG, Stokes MG (2017) Dynamic hidden states underlying working-memory-guided behavior. Nat Neurosci 20:864–871. 10.1038/nn.4546 28414333 PMC5446784

[B68] Woods DL, Alain C, Covarrubias D, Zaidel O (1995) Middle latency auditory evoked potentials to tones of different frequency. Hear Res 85:69–75. 10.1016/0378-5955(95)00035-3 7559180

[B69] Yi HG, Xie Z, Reetzke R, Dimakis AG, Chandrasekaran B (2017) Vowel decoding from single-trial speech-evoked electrophysiological responses: a feature-based machine learning approach. Brain Behav 7:e00665. 10.1002/brb3.665 28638700 PMC5474698

[B70] Zanto TP, Pan P, Liu H, Bollinger J, Nobre AC, Gazzaley A (2011) Age-related changes in orienting attention in time. J Neurosci 31:12461–12470. 10.1523/JNEUROSCI.1149-11.2011 21880908 PMC3205974

[B71] Zoefel B, VanRullen R (2017) Oscillatory mechanisms of stimulus processing and selection in the visual and auditory systems: state-of-the-art, speculations and suggestions. Front Neurosci 11:296. 10.3389/fnins.2017.00296 28603483 PMC5445505

